# Sciatica Cured by Chloroform

**Published:** 1850-09

**Authors:** John B. Holmes

**Affiliations:** Philadelphia


### ARTICLE XI.

*A Case of Sciatica Successfully Treated with Chloroform.*—By JOHN B. HOLMES, M. D., Philadelphia.

On the 17th of last October, Mrs. Scanlon, a poor woman seeking medical advice, called upon me. From a minute inquiry into her past and present life and sufferings, I gathered the following particulars: Mrs. Scanlon is a native of London—was born in the Winter of 1809. Her mother had five healthy children and three abortions; she was poor and compelled to labor hard at the wash-tub. Four years after the birth of Mrs. Scanlon, and immediately after her last abortion, she was attacked with a disease that baffled all efforts of the physician to either conquer or alleviate. This woman died in 1818, having suffered five years, during the last two of which she was bed-ridden. From the symptoms described by her daughter, and from other reasons which will hereafter appear, I believe she labored under Sciatica. A few years later, my patient removed to this country with the remainder of her mother's family, and at the age of 25 married. As time rolled on, she also became the mother of five healthy children, and, like her parent, unfortunately aborted three times. Then, and not till then, was her health impaired. Her system had received such a terrible shock as to incapacitate her for the duties of life, and cause her to pray not unfrequently for death. During the summer of 1848, her last pregnancy and abortion took place, after which she lay at the door of death nigh three weeks, and was ushered into this world again, as it were, but to suffer still greater torments. Since that time she has been tormented for fifteen months, first by Tic Doloureux and more recently by Sciatica. In this latter condition did she apply to me for aid and relief. I must confess that I was not a little surprised on comparing this, her story, with what appeared to me a picture of health, but such was nevertheless the case. And I am now thoroughly convinced that man may suffer cruel agony for months, nay years, without any inroad into the system that is appreciable.

Several physicians of this city attended Mrs. S. without affording her relief, or even mitigating her sufferings in the slightest degree. I commenced the treatment with the inhalation of Chloroform; but she had not applied the inhaler for more than one minute when she fell back, apparently dying, with the eyes turned upward, the pupils dilated, falling of the jaw, heaving and violent throbbing of the chest; but no inspiration or expiration resulted from the movement; her pulse was also imperceptible, and I soon found that spasm of the glottis had taken place. Instantly I threw cold water on her face, head and neck, and taking a bottle of Tinct. Camphor which lay upon the table, I poured a portion of the contents into her mouth, and held it to her nose. She soon commenced to revive, and in a short time I had the satisfaction of seeing her entirely relieved. A few days after this I administered 20 drops of Chloroform in ℥j. of Orgeat Syrup (which holds this preparation in admirable suspension) and found it productive of good. This dose was continued for 11 days, when I increased it to 25 drops. At the end of three weeks, finding her steadily improving. I thought it expedient to augment the dose still more, and boldly ordered 80 drops every two days. Seven weeks had elapsed since Mrs. S. had commenced the use of Chloroform, and so much relief was experienced that the medicine was discontinued. During the last two weeks of the above seven, she had but two attacks, lasting from 1 to 2 hours each. On the 10th of Dec., she called upon me and said her sufferings for twelve hours during the previous day had been great, and that she was then in pain. Recourse was had at once to Chloroform, and doses of 50 drops daily were continued a few days (five, I think), since which period she has been quite well, save a twinge or two during the damp weather of December and January.

